# Accuracy of CNV Detection from GWAS Data

**DOI:** 10.1371/journal.pone.0014511

**Published:** 2011-01-13

**Authors:** Dandan Zhang, Yudong Qian, Nirmala Akula, Ney Alliey-Rodriguez, Jinsong Tang, The Bipolar Genome Study, Elliot S. Gershon, Chunyu Liu

**Affiliations:** 1 Department of Pathology, School of Medicine, Zhejiang University, Hangzhou, People's Republic of China; 2 Department of Psychiatry and Behavioral Neuroscience, The University of Chicago, Chicago, Illinois, United States of America; 3 Intramural Research Programs, The National Institute of Mental Health, Bethesda, Maryland, United States of America; 4 Institute of Mental Health, The Second Xiangya Hospital, Central South University, Changsha, People's Republic of China; University of Miami, United States of America

## Abstract

Several computer programs are available for detecting copy number variants (CNVs) using genome-wide SNP arrays. We evaluated the performance of four CNV detection software suites—Birdsuite, Partek, HelixTree, and PennCNV-Affy—in the identification of both rare and common CNVs. Each program's performance was assessed in two ways. The first was its recovery rate, i.e., its ability to call 893 CNVs previously identified in eight HapMap samples by paired-end sequencing of whole-genome fosmid clones, and 51,440 CNVs identified by array Comparative Genome Hybridization (aCGH) followed by validation procedures, in 90 HapMap CEU samples. The second evaluation was program performance calling rare and common CNVs in the Bipolar Genome Study (BiGS) data set (1001 bipolar cases and 1033 controls, all of European ancestry) as measured by the Affymetrix SNP 6.0 array. Accuracy in calling rare CNVs was assessed by positive predictive value, based on the proportion of rare CNVs validated by quantitative real-time PCR (qPCR), while accuracy in calling common CNVs was assessed by false positive/false negative rates based on qPCR validation results from a subset of common CNVs. Birdsuite recovered the highest percentages of known HapMap CNVs containing >20 markers in two reference CNV datasets. The recovery rate increased with decreased CNV frequency. In the tested rare CNV data, Birdsuite and Partek had higher positive predictive values than the other software suites. In a test of three common CNVs in the BiGS dataset, Birdsuite's call was 98.8% consistent with qPCR quantification in one CNV region, but the other two regions showed an unacceptable degree of accuracy. We found relatively poor consistency between the two “gold standards,” the sequence data of Kidd *et al*., and aCGH data of Conrad *et al*. Algorithms for calling CNVs especially common ones need substantial improvement, and a “gold standard” for detection of CNVs remains to be established.

## Introduction

Copy number variation (CNV) is loosely defined as a deletion, duplication or inversion of a DNA sequence longer than one kilobase. CNVs have recently attracted considerable interest as a source of genetic variation because they may play an important role in the etiology of complex diseases and in evolution [1]–[14]. Because association studies of CNV and disease have become popular, genome-wide oligonucleotide arrays are now designed to detect both single nucleotide polymorphisms (SNPs) and CNVs [15]. The Affymetrix Genome-Wide Human SNP Array 6.0, for example, includes 906,600 SNP probes and 940,000 CNV probes.

A number of computer programs have been designed to detect CNVs using the intensity of the hybridization of sample DNA to the array probes. The underlying detection algorithm types are generally Hidden Markov model (HMM) [16], [17], or circular binary segmentation (genomic segmentation) [18]. PennCNV and QuantiCNV were first developed on an HMM-based algorithm for an Illumina platform [17], [19], then later modified to be compatible with Affymetrix platforms as well. Birdseye, another HMM-based approach, was developed to detect CNVs in SNP genotyping arrays specifically for Affymetrix platforms [16]. Two commercial software, Partek (Partek Inc., St. Louis, MO) and HelixTree (Golden Helix, Inc.), have implemented circular binary segmentation method.

Baross *et al* found considerable variation among the outputs from different programs, as well as substantial false call rates for CNVs, when they compared four CNV detection programs [20]: Copy Number Analyser for GeneChip® arrays (CNAG) [21], DNA-Chip Analyzer (dChip) [22], Affymetrix GeneChip® Chromosome Copy Number Analysis Tool (CNAT)[20], [23] and Gain or Loss Analysis of DNA (GLAD) [24]. Winchester *et al*. reviewed 12 programs and assessed 7 of them using published CNV data in HapMap samples [25]. The programs they assessed were Birdsuite, CNAT, Genome Alteration Detection Algorithm (GADA) [26], PennCNV, QuantiSNP, CNVPartition (Illumina Inc. CA), and Nexus (BioDiscovery, Inc. CA). Like the other papers, they observed large variation among different programs as well as different platforms (Illumina vs. Affymetrix). However, Winchester *et al* did not draw any conclusions on the performance of the programs, and it is difficult to judge which program was superior based on the data they provided.

Korn *et al* compared their program, Birdsuite, with two commercial tools, Partek and Nexus [16]. They tested the three programs using eight HapMap samples in which 893 CNVs had been previously identified by Kidd *et al* using fosmid end-pair sequencing (EPS) and validated by array comparative genomic hybridization or full-length resequencing [13]. They calculated and compared the rates at which the programs recovered these CNVs [16]; a reference CNV was considered recovered if the program called a CNV that shared at least 25% of the length spanned by the reference and called CNV together. The recovery rate of the three programs ranged from 11.6% for Partek to 93.8% for Birdsuite for regions containing more than 20 probes. However, note that Birdsuite was originally developed on HapMap data.

None of the previous studies evaluated rare and common CNV calls separately, even though common CNVs are more difficult to call accurately than rare ones. This difference may arise because some programs use all the samples in a given batch to create a reference signal at a given SNP, to which individual signals from that batch can be compared. For example, HelixTree and PennCNV-Affy establish a reference in this way, then use the log_2_ of the ratio of the hybridization intensity of an individual subject to the intensity of the reference (log_2_ R (subject/reference)) for further segmentation or for modeling copy number status [17]. As a result, frequently-occurring variations make it harder to establish a true reference signal. The same problem occurs in Partek if the option to use the log_2_ ratio for segmentation is selected.

In the Birdsuite package, on the other hand, the Canary program is designed to call common CNVs previously established by McCarroll *et al* [15]. The designers constructed a series of prior models based on summarized intensity measurements of HapMap samples with different copy number states for the established common CNVs. This approach avoids the common variant reference problem, but it is still problematic because the HapMap sample is not necessarily an appropriate reference for a given study population. For rare or *de novo* CNVs, Birdsuite has an entirely different program, Birdseye, which establishes a reference intensity similar to that of HelixTree or PennCNV-Affy. It extracts the intensity of all samples in a given batch, then excludes those regions already determined to be copy-variable via Canary, and also excludes the 10% of samples with the highest intensities and the 10% of samples with the lowest intensities [16]. It uses the results to estimate the mean and variance of the normal distribution of two copies.

In this study, we assessed four currently used CNV detection software programs for their accuracy in detecting both rare and common CNVs in the Affymetrix 6.0 platform. We used Affymetrix SNP Array 6.0 data in 270 HapMap samples and 1001 bipolar cases and 1033 controls of European ancestry from Bipolar Genome Study (BiGS). The software packages tested were Birdsuite (version 1.5.2), PennCNV-Affy (a trial version), HelixTree (Version 6.4.2), and Partek (Version, 6.09.0129). We assessed their recovery rate per Korn *et al*'s method with modifications as described below [16]. In addition, based on qPCR, we estimated their false positive and false negative call rates for three common CNVs, as well as their positive predictive values for singleton CNVs, i.e., those that occur once in a dataset.

## Results

### Recovery test based on sequencing data of eight HapMap samples

We used the same 893 CNVs used by Korn *et al*. as a reference to compare the recovery rates of Birdsuite, Partek, PennCNV-Affy and HelixTree [16]. Korn *et al*.'s Birdsuite recovered CNVs spanning more than 20 markers at a rate (88.5%) comparable to that of Korn *et al* (93.8%) when using their criteria (File S1: Table S1) [16]. With our additional requirement of copy number consistency (the CNV status identified by the programs should be in the same direction as the reference CNV), the recovery rate of Birdsuite decreased from 88.5% to 70%, whereas PennCNV-Affy, Partek and HelixTree had relatively smaller decreases (5–6%). Consistent with the results of Korn *et al* [16], the recovery rate increased with the number of probes spanned by the CNV for all algorithms (Table 1). The sensitivity of PennCNV-Affy increased from 55.4% to 58.5% when pedigree information was used, which is a unique option of PennCNV-Affy.

**Table 1:** The recovery rates of each of the CNV-calling programs depending on CNV length based on data of Kidd et al.

# markers	# CNVs in the reference list from *Kidd et al*.	# CNVs recovered by Birdsuite	# CNVs recovered by Partek	# CNVs recovered by PennCNV-Affy_trios*	# CNVs recovered by PennCNV-Affy	# CNVs recovered by HelixTree
1	329	6 (1.8%)	0	0	0	2 (0.6%)
2–5	249	71 (28.5%)	0	3 (1.2%)	2 (0.8%)	19 (7.6%)
6–10	112	47 (42.0%)	10 (8.9%)	20 (17.9%)	11 (9.8%)	28 (25%)
10–20	73	32 (43.8%)	26 (35.6%)	27 (37.0%)	24 (32.9%)	17 (23.3%)
>20	130	91 (70.0%)	70 (53.8%)	76 (58.5%)	72 (55.4%)	54 (41.5%)

*The pedigree information was incorporated with the calling of CNVs.

Recovery rate was calculated with the requirement of copy number consistency.

Closer inspection of the frequency of 130 CNVs (112 regions containing more than 20 markers) revealed that 100 of these CNV regions occurred only once in the eight HapMap samples. The recovery rate of individual CNVs decreased as the frequency of the CNVs increased (File S1: Figure S1). We further looked at the size of CNVs that spanned more than 20 markers and calculated the recovery rate in each size bin. There was no correlation between the size and the recovery rate when the analysis was limited to CNVs containing more than 20 markers (data not shown).

### Recovery test based on array CGH data of 90 CEU HapMap samples

We used 51,440 CNVs detected and validated in 90 CEU samples by Conrad *et al* as a reference to compare the recovery rate of Birdsuite, Partek, PennCNV-Affy and HelixTree [14]. The recovery rate also increased with the number of probes spanned by the CNV for all algorithms (Table 2). However, the highest recovery rate for any program on detection of CNVs with >20 markers was only 47.69% by Birdsuite.

**Table 2:** The recovery rates of each of the CNV-calling programs depending on CNV length based on data by Conrad *et al*. in 90 CEU samples.

# markers	# CNVs in the reference list from Conrad *et al*. *	# CNVs recovered by Birdsuite	# CNVs recovered by Partek	# CNVs recovered by PennCNV-Affy_trios**	# CNVs recovered by HelixTree
1	28366	88 (0.31%)	2 (0.007%)	111 (0.39%)	110 (0.39%)
2–5	11837	1362 (11.51%)	8 (0.068)	138 (1.17%)	486 (4.11%)
6–10	3142	926 (29.47%)	209 (6.65%)	599 (19.06%)	720 (22.92%)
10–20	2754	973 (35.33%)	507 (18.41%)	747 (27.12%)	711 (25.82%)
>20	5341	2547 (47.69%)	1400 (26.21%)	1883 (35.26%)	1770 (33.14%)

*5,341 CNVs spanned by >20 markers were included in this analysis.

**The pedigree information was incorporated with the calling of CNVs.

We further calculated the average recovery rate of CNVs with different frequency spanned by more than 20 makers. The average recovery rate decreased with the increased frequency of CNVs. The average recovery rate of CNVs with a frequency ≤20% ranged from 65.62% by Partek to 85.95% by PennCNV-Affy with pedigree information incorporated (See Table 3). CNVs with a frequency larger than 80% were difficult to recover (average recovery rate: 10.81%–30.50%).

**Table 3:** The average recovery rates of each of the CNV-calling programs depending on CNV frequency based on data by Conrad *et al*. in 90 CEU samples.

Frequency(a)	# CNVs in the reference list from Conrad *et al*.*	# CNVs recovered by Birdsuite	# CNVs recovered by Partek	# CNVs recovered by PennCNV-Affy_trios**	# CNVs recovered by HelixTree
a< = 20%	669	537 (80.27%)	439 (65.62%)	575 (85.95%)	528 (78.93%)
20%<a< = 40%	488	270 (55.33%)	221 (45.29%)	329 (67.41%)	314 (64.34%)
40%<a< = 60%	793	579 (73.01%)	210 (26.48)	373 (47.04%)	442 (55.74%)
60%<a< = 80%	765	352 (46.01%)	105 (13.73%)	139 (18.17%)	123 (16.08%)
80%<a< = 1	2626	801 (30.50%)	284 (10.81%)	366 (13.94%)	342 (13.02%)

*CNVs spanned by more than 20 markers were included in this analysis.

**Pedigree information was incorporated.

CNV regions spanned by more than 20 markers were compared across programs. Birdsuite, PennCNV-Affy and Helixtree can recover about 50% of CNV regions with a sensitivity ≥90% and 30% of CNV regions with a sensitivity ≤10%. For example, Birdsuite recovered 58.21% of CNV regions with a very high sensitivity (>90%), and 34.83% regions with a sensitivity ≤10%. The percentage of CNV regions, with a recovery rate higher than 90%, decreased from 66.39% to 28.13% when the frequency of CNVs increased from ≤20% to >80% (File S1: Table S2). 21 highly frequent (>80%) but poorly recovered (≤10%) CNVs were all duplications. Seven out of nine highly frequency regions with high recovery rates were deletions (File S1: Table S2, Birdsuite data).

Separately, we compared the consistency of the CNVs reported by Kidd *et al* and by Conrad *et al* in the same 8 HapMap individuals. Two CNVs identified by Conrad *et al* and Kidd *et al* were considered as the same if they shared at least 25% of the total length spanned. 3,024 out of 4,537 CNVs (66.65%) from Conrad *et al* were not larger than 5 kb while 89.33% of CNVs from Kidd *et al* were larger than 10 kb. This is expected because of the greater density of probes in Conrad *et al*. But overall, there was not an impressive degree of overlap between the two studies, even for the larger CNVs (Table 4). Yet each of these studies has been considered as a potential “gold standard” for CNV calling.

**Table 4:** CNVs detected in 8 HapMap individuals shared between two studies.

Size (kb)	# of CNVs in Conrad *et al* (total 4,537)	# detected by Kidd *et al* (Percentage) *	# of CNVs in Kidd *et al* (total 9,513)	# detected by Conrad *et al* (Percentage)
≤5	3024	38 (1.27%)	441	1 (0.23%)
5to 10	647	149 (23.02%)	574	13 (2.26%)
10 to 50	514	146 (28.40%)	8174	300(3.67%)
50 to 100	180	19 (10.56%)	217	39 (17.97%)
100 to 1000	172	11 (6.40%)	107	11 (10.28%)

*Criterion: Two CNVs from Conrad *et al* and Kidd *et al* were considered as the same if they shared at least 25% of the total length spanned. One CNV from Conrad *et al* can share with more than one CNV from Kidd *et al*, vice versa.

### Evaluation of CNV calls in the BiGS data set

**Figure 1:**
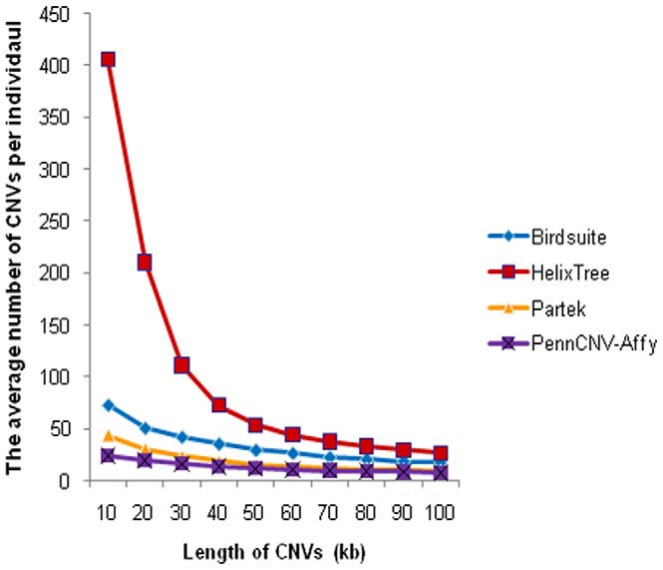
The average number of CNVs per individual called by each of the four CNV-calling programs. The average number of CNVs per individual varies greatly among programs, especially for CNVs less than 10 kb. The X axis represents length of CNVs. The Y axis is the average number of CNVs per individual.

The average number of CNVs called per individual varied greatly among programs (Figure 1); for example, for CNVs larger than 100 kb, PennCNV-Affy called an average of 8, while HelixTree called an average of 27. The differences declined when the size of CNVs increased, so when selecting CNVs for qPCR validation, we chose CNVs larger than 100 kb.

### Singleton deletions and duplications in the BiGS data set

The largest number of program-specific singleton deletion calls was made by HelixTree (Table 5), at a surprisingly higher frequency than Birdsuite, Partek and PennCNV-Affy, which tended to agree with each other. Although software agreement does not necessarily correspond with the validity of the calls, the program-specific calls tended not to be validated, as demonstrated below. More singleton duplications were identified by each program than singleton deletions (Table 5); however, substantial variation among programs was observed in the number of singleton duplications. HelixTree produced the highest percentage of program-specific singleton duplications.

**Table 5:** The number of singleton CNVs called by each program, and percentage by which they overlap with calls made by the other programs.

	Program	Birdsuite	HelixTree	Partek	PennCNV-Affy
Deletions	Shared **	306(90.5%)*	250(38.8%)	279(90.0%)	228(80.6%)
	Program-specific ***	32(9.5%)	394(61.2%)	31(10.0%)	55(19.4%)
	Total	338	644	310	283
Duplications	Shared	401(74.7%) *	332(41.7%)	354(90.8)	289(85.3%)
	Program-specific	136(25.3%)	465(58.3%)	36(9.2%)	50(14.7%)
	Total	537	797	390	339

*Data format: number of events (percentage of events shared by other programs).

**Shared singleton deletions or duplications were defined as CNVs called by one program that overlapped at all with singleton deletions or duplications called by any other program.

***Program-specific CNVs are those that did not overlap at all with any singleton deletions or duplications called by any other program.

### qPCR validation of selected singleton CNVs in the BiGS data set

The positive predictive values of Birdsuite and Partek for the sampled deletions were 100%. This finding agrees with our previous finding of a positive predictive value for Birdsuite using 19 singleton deletions [10]. Strikingly, none of the five sampled deleted regions called by HelixTree were confirmed by qPCR. Three out of five deleted regions called by PennCNV-Affy were confirmed as deletions by qPCR, a positive predictive value of 60% (Table 6).

**Table 6:** Positive predictive value for rare CNVs of each program, based on qPCR validation of their program-specific singletons.

Programs	Birdsuite	HelixTree	Partek	PennCNV-Affy
Deletions	5/5 = 100%*	0/5 = 0%	5/5 = 100%	3/5 = 60%
Duplications	2/5 = 40%	2/5 = 40%	2/6 = 33.3%	4/6 = 66.7%

*Positive predictive value: true positive/(true positive + false positive). For each region, five samples were tested, one with a putative deletion/duplication, the other four with two putative).

For each program, we also randomly selected five to six program-specific singleton duplications for qPCR validation. None of the programs had all the selected program-specific duplications validated: positive predictive values ranged from 40% to 66.7%.

### Common CNVs in the BiGS dataset

We observed that unlike rare CNV calls, common CNVs calls were influenced by plate effects. Common CNVs called by Birdsuite's Canary program were tested for plate effects by doing a series of GWASs plate by plate. We compared the CNV frequencies measured for each plate against those of all other plates in each analysis, using PLINK. About 44% of common CNVs showed a plate effect (data not shown).

Three common CNV regions frequently called by Canary were randomly selected for qPCR investigation: CNP1293, which showed no plate effect, and CNP2157 and CNP2057, which both had plate effects; these CNVs are located on chr8:39354760-39506122 (56 CN and 3 SNP probes), chr16:22465433-22612022 (61 CN and 16 SNP probes), and chr15:19803370-20089386 (104 CN and 60 SNP probes), respectively. The frequencies of duplication and deletion of these three regions as called by each program are shown in Table 7.

**Table 7:** Frequencies with which each program calls three common CNVs identified by Canary in the BiGS dataset.

ID		Birdsuite	HelixTree	Partek	PennCNV-Affy
CNP2157	Frequency of Duplications	1833(90.1%)	29 (1.4%)	24 (1.2%)	15 (0.7%)
	Frequency of Deletions	1 (0.05%)	187 (9.2%)	42 (2.1%)	38 (1.9%)
CNP1293	Frequency of Duplications	1 (0.05%)	664 (32.6%)	619 (30.4%)	508 (25.0%)
	Frequency of Deletions	1277(62.8%)	449 (22.1%)	344 (16.9%)	262 (12.9%)
CNP2057	Frequency of Duplications	170(8.4%)	254 (12.5%)	197 (9.7%)	103 (5.1%)
	Frequency of Deletions	653(32.1%)	248 (12.2%)	142 (7.0%)	188 (9.2%)

To validate these CNV calls, we validated 3 CNPs with qPCR. We assayed 69 samples with qPCR for CNP2157 and found that CNV calls by all of the programs in this region were imprecise (Table 8). For CNP2057, we quantified 87 samples; none of the programs performed satisfactorily. When 85 samples were studied for CNP1293, which had no plate effect, we found that, strikingly, Birdsuite achieved a 0% false positive rate and a 1.9% false negative rate, while the other three programs all had a low sensitivity (i.e., 1 - the false positive rate) and specificity (i.e., 1 - the false negative rate) for this CNV. The performance of each program varied greatly from CNV to CNV; none performed consistently. The results observed here were consistent with the recovery rate evaluated in HapMap samples especially for Birdsuite (see above).

**Table 8:** qPCR validation of the calls made by each program for three common CNVs identified by Canary in the BiGS dataset.

CNP		Birdsuite	HelixTree	Partek	PennCNV-Affy
CNP2157	False positive rate	100%	8.5%	0.0%	0.0%
	False negative rate	100%	66.7%	66.7%	66.7%
CNP1293	False positive rate	0.0%	96.9%	71.9%	71.9%
	False negative rate	1.9%	80.8%	80.8%	80.8%
CNP2057	False positive rate	55.1%	0.0%	0.0%	0.0%
	False negative rate	62.5%	46.9%	62.5%	62.5%

## Discussion

The sensitivity and specificity of CNV identification is an essential component of association studies of CNVs with disease. In our evaluation of two commercial programs and two publicly available programs widely used for CNV identification in GWAS data, we found considerable variation among the programs in the number of CNVs called. The differences declined when the size of CNVs increased, but substantial variation still existed in the accuracy of CNV calls as determined by the recovery test and by qPCR validation, even for large CNVs containing more than 20 markers or larger than 100 kb in length.

For recovery of CNVs detected by Korn *et al* in eight HapMap samples, Birdsuite was superior to the other three programs, as it recovered more CNVs in each category. However, the sensitivity of all four programs was poor when the number of probes spanned by a CNV was small. For CNVs containing more than 20 markers, Birdsuite recovered 88.5% of CNVs, which is comparable to Korn *et al*'s finding of 93.8% for Birdsuite [16]. Some of the discrepancy may be explained by the fact that Korn *et al*. analyzed the same samples but with different. CEL files produced by different labs; the files used in the present study were obtained from Affymetrix, while Korn *et al*. used. CEL files produced in their own lab [16]. Also, their Birdsuite recovery rate was inflated since no agreement on CNV state (deletion or duplication) was required by Korn *et al*. just after the development of Birdsuite; we found that only 70% of CNVs containing 20 or more markers were recovered by Birdsuite with an agreement on CNV state.

Birdsuite's recovery rate of common CNVs containing more than 20 markers varied across genomic regions. For example, there were eight CNV regions carried by any two of the eight HapMap individuals, and therefore common. Six of those eight regions were recovered in both individuals by Birdsuite, but the other two regions were not recovered. Poor recovery rates of CNVs with high frequency (i.e., carried by more than three of the eight HapMap samples), was also observed (File S1: Figure S1).

As expected, the sensitivity of Partek showed a 5.2-fold increase, to 60.7%, after using quantile normalization, as compared to Korn *et al*.'s finding of 11.2% without using quantile normalization. A very recent study has shown that normalization can improve performance in analysis of miRNA array data and that quantile normalization is the most robust normalization method [27]. Like all microarrays, Affymetrix SNP arrays are affected by systematic sources of experimental variation. Normalization can help reduce or remove noise that distorts the distribution of observed array data, and thus improve the accuracy of genotyping calls and copy number calls.

For our recovery test in 90 CEU HapMap samples, the highest recovery rate was only 47.91% from Birdsuite, in detecting CNVs spanned by more than 20 markers. Consistent with the recovery rate shown above, the average recovery rate decreased with increased CNV frequency. On closer inspection, it is clear that Birdsuite's recovery rate of most CNVs containing more than 20 markers was either ≤10% or >90% (File S1: Table S2). For example, there were 32 CNV regions that showed a very high frequency in CEU HapMap samples (>80%). Six out of 32 CNV regions were 100% recovered but the recovery rates of 21 CNV regions were less than 10%, which is consistent with our qPCR results on tested common CNVs. Regardless the recovery rate, the number of CNVs identified by different programs varied greatly. The most CNVs were identified by Helixtree, almost 4 times the number of CNVs detected by Birdsuite and 7 times that of CNVs detected by PennCNV-Affy and Partek.

One possible reason for the discrepancy of recovery rate in two datasets (Kidd *et al* and Conrad *et al*) is that the detection method: very dense microarrays used by Conrad *et al* vs. sequencing used by Kidd *et al* [13], [14]. Conrad *et al*'s experiment design aimed to discover CNVs of size greater than 500 bp. However, the median size of insert clone is around 40 kb in paired-end sequencing by Kidd *et al*., which would make detection of small CNVs more challenging. Consequently, more small CNVs (≤5 kb) were validated by Conrad *et al*, and more median size CNVs (10–50 kb) were reported by Kidd *et al*. This may also partially explain the poor consistency rate of CNVs between the two studies.

There was significant variation in singleton deletion calls among programs: HelixTree detected about two-fold more singleton deletions in the BiGS data set (644 singleton deletions) than Birdsuite, Partek, or PennCNV-Affy. However, more than half of those detections were specific to HelixTree (59.3%), and the majority of HelixTree program-specific calls of deletions were not validated by qPCR. This meant that for program-specific singleton deletions, the positive predictive value of HelixTree was zero based on the qPCR validation of sampled regions. For Partek and Birdsuite, on the other hand, all selected singleton deletions were validated by qPCR. In the recovery test and in qPCR results on singletons, Birdsuite had the best performance among the tested programs.

We checked closely the program-specific calls because some authors have proposed to combine CNV calls from multiple independent software calls to improve accuracy. The program-specific calls in our data tended not to be validated (most likely to be in error). Positive predictive value could not be calculated in an unbiased manner in the present study since we only selected program-specific CNVs for qPCR validation. A sampling from all CNVs (both program-specific and shared calls) would provide a more accurate measure of predictive values.

More singleton duplications were called by each program in the BiGS dataset than singleton deletions, and each program called singleton duplications that were not called by any of the other three programs. The average positive predictive value for these program-specific singleton deletions for all four programs (65%) was higher than for singleton duplications (45%) in our tested regions. We speculate that the performance of programs in the detection of deletions is better than the detection of duplications because a deletion represents a 2-fold change in copy number while a duplication produces only a 1.5-fold change. As might be expected, 21 highly frequent (>80%) but poorly recovered (≤10%) CNVs were all duplications (File S1: Table S2 Birdsuite's recovery data).

For common CNV regions identified by Canary in the present study, we observed striking variation in the frequency of calls by different programs. Each program had substantial false positive and false negative rates in at least one of the three CNV regions tested. The high false positive and negative rates observed here may be due to the fact that common CNVs will affect the mean and variance of hybridization intensity over the region included and thus affect the observed log2 ratio of the CNV. The fact may also lead to the poor recovery rate of highly frequent CNVs.

Plate effects may play a significant role in the accuracy of common CNVs called by Canary. CNP1293, which showed no plate effect, had a high sensitivity and specificity, but CNP2157 and CNP2057 had plate effects and showed a very low sensitivity and specificity. According to our limited qPCR results, the algorithms evaluated here for calling common CNVs all need improvement. We would conclude that without independent experimental genotyping, software-called common CNVs based on GWAS array data are not suitable for association studies. In contrast, rare CNVs called by Birdsuite and Partek are of substantially better quality. Similarly, Marenne *et al* concluded that further validation was required to assess CNVs as risk factors in complex diseases when they evaluated CNVpartition, PennCNV and QuantiSNP using Illumina Infinium Human 1 Million SNP array data[28].

Recently, Mei *et al* developed two new methods to identify common CNV regions [29]. They evaluated their methods with sequencing-based results from Kidd *et al*. However, the lowest discordance rate was 55% after excluding individual regions with a confidence score (as developed by them) below the 80^th^ percentile. Two previously published methods, STAC and GISTIC had similar performance at identifying CNVs with high frequency and moderate confidence [30], [31]. These reports further confirmed our observation that common CNV detection methods still have much room for improvement.

Winchester *et al* recommended using a second program to generate the most informative results [25]. This recommendation seems to be based on the assumption that the second program performed similarly to the first one, and that their overlap increases the reliability. This might not be a safe assumption when not all software suites perform equally well. Birdsuite is a better choice for identifying rare CNVs than the others in our evaluation.

One limitation of the present study is the small number of CNVs tested by qPCR, particularly for the common CNVs. Although the number of qPCR tests performed for validation was limited, the overall trend of frequent non-validation is in agreement with other results from larger datasets (the recovery tests on HapMap samples). These two independent lines of evidence support our concerns regarding the validity of CNV calls based on GWAS data.

The intention of this study is to identify potential traps of current practice in the GWAS-based CNV analysis, rather than an attempt to provide a solution. It is possible that program tweaking would improve accuracy, but it appeared reasonable to start with the default parameters recommended by each program's provider.

We evaluated the reproducibility of the two “gold standards” used in this study, the paired-end sequence data of Kidd *et al*, and the very high density array Comparative Genomic Hybridization (aCGH) in the same 8 HapMap individuals. We found relatively poor consistency between the two “gold standards.” The lack of a standard sets a limit on much of the recovery of GWAS-based CNV calls, particularly for common CNVs since they would be over-represented when 8 individuals are studied. Next-generation sequencing of whole genome of population samples might be able to provide an ultimate gold standard for identification of common CNVs.

A more extensive list of independently validated CNV regions, and the raw hybridization or other data files used to detect them, should be made publicly available. A greatly expanded version of Kidd *et al*'s or Conrad *et al*'s HapMap data set used in this and previous studies [13], with all CNVs confirmed by high coverage sequencing, and with the addition of parental data, might provide an acceptable resource. The public data from dbGaP and similar sources can also be used for this purpose, as can the CNV validation data we have produced in this study and plan to produce in future studies. Once these datasets are available, independent validation studies must be performed, even though they require the expenditure of valuable time and funds.

## Materials and Methods

### Subjects

To test the programs' recovery rates, we obtained the Affymetrix Genome-Wide Human SNP Array 6.0 data (SNP Array 6.0) from 270 HapMap samples (90 CEPH, 90 YRI and 45 CHB and 45 JPT) from Affymetrix. To test the programs' false positive/negative rates for common CNVs and positive predictive values for singletons, we obtained Bipolar Genome Study (BiGS) genotype data from 1001 bipolar cases and 1033 controls of European ancestry via dbGaP (phs000017.v1.p1); those data were collected using the Affymetrix Genome-wide Human SNP Array 6.0, details described elsewhere [32].

### Software algorithms and parameters

We performed plate-wise quantile normalization, then identified CNVs for each plate using each of the four programs. The settings of the software packages are presented in Table 9. Since the optimal parameters for a given dataset may not be optimal for another dataset, we chose to mainly use the programs' default parameters to make the comparisons as fair as was possible. To better compare HelixTree and Partek, log2 ratios were created by normalizing raw intensity data against a set of reference samples from a given batch in HelixTree, and then were called by Partek and HelixTree for CNVs using segmentation. Genomic segmentation was recommended by Partek and was therefore used for evaluation here. For PennCNV-Affy, we ran the program with and without the pedigree information.

**Table 9:** Settings used for each of the four software suites.

Software	Plate-wise quantile normalization	Detecting algorithm	Parameters
Birdsuite	Yes (APT)*	HMM	Using population-specific prior models***
HelixTree	Yes (HelixTree)	Segmentation	Default
Partek	Yes (HelixTree) **	Segmentation	Default
PennCNV-Affy	Yes (APT)	HMM	No prior models

For HelixTree and Partek, default settings were used; normalization was done before CNV calling. For PennCNV-Affy, the standard procedure was followed without wave adjustment. For Birdsuite, population-specific prior models were employed.

* Platewise normalization was done by Affy Power Tools (APT1.10.0) plug in Birdsuite/PennCNV-Affy.

** Normalization was done by HelixTree.

*** For Canary, the appropriate prior model was selected based on the ancestry of the sample.

### Measuring CNV recovery rates in eight and 90 CEU HapMap samples

For eight HapMap samples, we used the same recovery statistic as Korn *et al*., with one modification. Since deletions identified by one program could be called as duplications by another program, an additional criterion was included that not only a variation be called, but that it should be in the same direction as the reference CNV, i.e., a CNV called as 0 copy or 1 copy had to correspond to a deletion called in the eight validated HapMap samples in Kidd *et al* [13]. If it did not match, the reference CNV was not considered recovered. Similarly, another recovery rate was calculated based on 51,440 reference CNVs validated in 90 CEU samples reported by Conrad *et al* [14]. Normalization was done prior to segmentation when evaluating Partek. We used the same inclusion criterion for CNV calls as Korn *et al* [16], requiring CNV calls to have LOD scores (probability of the segment being the stated copy number versus the copy number of the flanking region) ≥5 for inclusion.

### Consistency of CNVs Detected in 8 HapMap Individuals in Two Studies (Conrad et al and Kidd et al)

CNVs validated by Conrad *et al* were compared with CNVs reported by Kidd *et al*. Two CNVs from Conrad *et al* and Kidd *et al* were considered as the same if they shared at least 25% of the total length spanned. One CNV from Conrad *et al* can share with more than one CNV from Kidd *et al*, vice versa.

### Comparison and validation of CNV calls in the BiGS dataset

In the BiGS dataset, all CNV calls made by all four programs were compared (Figure 1). Birdsuite is the only one of the four that filters out CNVs with LOD scores less than 10.

We are particularly interested in the role of singleton CNVs in common diseases [10], so when choosing rare CNVs to validate, we focused on singleton CNVs. Singletons were defined as deletions or duplications that occurred only once in the entire BiGS data set, including controls, and did not overlap with any other CNVs. The analysis was done by PLINK (version 1.05) [33].

To validate the singleton CNVs called by the software packages in the BiGS sample, quantitative real-time PCR (qPCR) with SYBR-green dye was used to measure the copy number of a subset of singleton CNVs using the ABI Prism® 7900HT Sequence Detection System. The copy number at target regions relative to the reference is approximately 2^− ΔΔCt^ (for more details see elsewhere [10]), where Ct, the threshold cycle number, is a function of the amount of starting template.

We selected a total of 20 singleton deletions and 22 singleton duplications for qPCR validation. For each program, five or six duplications and five deletions were randomly selected that were uniquely called by that program, which we refer to as program-specific calls. We then designed three pairs of primers for each region and tested five samples for singleton duplications and five other samples for singleton deletions. So, in each test, one sample carried a program-specific singleton deletion or duplication, and the other four samples did not carry any CNVs called that overlapped that region, i.e., all programs called two copies for that sample.

To validate the common CNVs called by the software packages for the BiGS sample, we randomly selected one common duplication and two common deletions called by Canary for qPCR validation. We used TaqMan® Copy Number Assays from ABI (Applied Biosystems Inc, CA) on the ABI Prism® 7900HT Sequence Detection System for validation since the target and an endogenous control can be amplified in the same reaction. Only one probe was designed for each region, so CNVs called by different programs were required to share at least 50% of the total length spanned by the two calls combined to be considered the same CNV. All the primers and probes designed for qPCR are provided in supplementary data (File S1: Table S3).

To assess the relative accuracy of the programs in detecting rare CNVs in the BiGS data set, we calculated and compared their positive predictive values, i.e., the ratio of true positives to positive calls both true and false; data to calculate false negative and false positive rates were not collected, since these CNVs are, by definition, rare. To compare the programs' abilities to detect common CNVs, we calculated and compared their specificities and sensitivities.

### Web resources

http://www.biodiscovery.com/index/nexus

http://www.goldenhelix.com

http://www.openbioinformatics.org/penncnv/penncnv_tutorial_affy_gw6.html

http://www.hapmap.org/

http://www.affymetrix.com

http://www.ncbi.nlm.nih.gov/gap

http://pngu.mgh.harvard.edu/purcell/plink/)

http://www3.appliedbiosystems.com/AB_Home/index.htm

http://www.partek.com

## Supporting Information

File S1 Includes Tables S1 to S3 and Figure S1.
(0.42 MB DOC)
