# Supplementary material for: Accuracy of CNV Detection from GWAS Data
Source: PLoS One. 2011 Jan 13;6(1):e14511. doi: 10.1371/journal.pone.0014511 (PMC3020939; doi:10.1371/journal.pone.0014511)

**Supporting Information** **S1**

**Table S1. Sensitivity of 4 programs without the requirement of the consistency of copy number type (duplication or deletion)**

| # markers | # CNVs in the reference  list from Kidd et al’s study | # CNVs recovered by Birdsuite | # CNVs recovered by Partek | # CNVs recovered by PennCNV-Affy_trios | # CNVs recovered by PennCNV-Affy | # CNVs recovered by HelixTree |
| --- | --- | --- | --- | --- | --- | --- |
| 1 | 329 | 11 (3.3%) | 2 (0.6%) | 2 (0.6%) | 2 (0.6%) | 4 (1.2%) |
| 2-5 | 249 | 77 (30.1%) | 1 (0.4%) | 4 (1.6%) | 2 (0.8%) | 20 (8.0%) |
| 6-10 | 112 | 54 (48.2%) | 10 (8.9%) | 20 (17.9%) | 11 (9.8%) | 28 (25.0%) |
| 10-20 | 73 | 43 (58.9%) | 29 (39.7%) | 31 (42.5%) | 26 (35.6%) | 19 (26.0%) |
| >20 | 130 | 115 (88.5%) | 79 (60.8%) | 84 (64.6%) | 78 (60.0%) | 60 (46.2%) |

**Table S2. The number of recovered CNV regions spanned by more than 20 markers in 90 CEU samples from Conrad *et al***

| Frequency (a) | Recovery rate (b) | # of CNV regions recovered by each program | | | |
| --- | --- | --- | --- | --- | --- |
|  |  | Birdsuite | Partek | PennCNV-Affy_trios | HelixTree |
| All CNVs | ≤0.1 | 70 (34.83%) | 61 (30.35%) | 50 (24.88%) | 58 (28.86%) |
|  | 0.1-0.9 | 14 (6.96%) | 82 (40.80%) | 48 (23.88%) | 58 (28.86%) |
|  | 0.9-1 | 117 (58.21%) | 58 (28.86%) | 103 (51.24%) | 85 (42.29%) |
| a≤20% | ≤0.1 | 31 (25.41%) | 27 (22.13%) | 19 (15.57%) | 25 (20.49%) |
|  | 0.1-0.9 | 10 (8.2%) | 40 (32.79%) | 11 (9.02%) | 24 (19.7%) |
|  | 0.9-1 | 81 (66.39%) | 55 (45.08%) | 92 (75.41%) | 73 (59.84%) |
| 80%<a≤1 | ≤0.1 | 21 (65.63%)* | 23 (71.88%) | 23 (71.88%) | 22 (68.75%) |
|  | 0.1-0.9 | 2 (6.25%) | 9 (28.13%) | 9 (28.13%) | 10 (31.25%) |
|  | 0.9-1 | 9 (28.13%)** | 0 (0.00%) | 0 (0.00%) | 0 (0.00%) |

*** 21 regions were all duplications.**

**** Seven out of nine regions were deletions.**

**Table S3. Primer sequences**

Primer information, table containing the primer sequence and the targeted CNV region location.

| **Primer names** | **Primer sequence** | **CNV regions targeted** | | |
| --- | --- | --- | --- | --- |
|  |  | **chr** | **start** | **end** |
| 16022_seq1-F | CAACGGAGACAATTTACATAATGCA | 3 | 50466298 | 50596947 |
| 16022_seq1-R | ACTAACGGGCACCCTGCTT |  |  |  |
| 16022_seq2-F | GCCCACAGACCTGGCCTAT |  |  |  |
| 16022_seq2-R | CATCTGGACATTTCCGAACCA |  |  |  |
| 16022_seq3-F | CCGTGCCTACCCCTTTCTC |  |  |  |
| 16022_seq3-R | TCGAGGTGGATGATCCAAATC |  |  |  |
| 17004_seq1-F | GCTGGTCTGGACAAGGGTCTAG | 11 | 538339 | 654854 |
| 17004_seq1-R | ACCCTGATCCTAAGTTAACACGCTAT |  |  |  |
| 17004_seq2-F | TGAGGCCGAGCTGAGGTTT |  |  |  |
| 17004_seq2-R | CCGGATGGTCCAGGAGAGA |  |  |  |
| 17004_seq3-F | CTCCTCTTTGGGATTTCATGGT |  |  |  |
| 17004_seq3-R | CCGGTCATTGGCTGGTATG |  |  |  |
| 19119_seq1-F | GGTGGCCAGAGATGAACCAAT | 10 | 102192619 | 102298204 |
| 19119_seq1-R | AGGCCTGCTCATTCCAGATG |  |  |  |
| 19119_seq2-F | GGCTTCGGAGAGGCGATT |  |  |  |
| 19119_seq2-R | CCGTGCATCCTGTCCTGTTT |  |  |  |
| 19119_seq3-F | TGAGCAGTGCATCTGTGAACAA |  |  |  |
| 19119_seq3-R | CCATTTCAACTGGGCAAAAGA |  |  |  |
| 29853_seq1-F | CCAGAGGAGACCTTGAAAATATAAACTC | 3 | 193726686 | 194360584 |
| 29853_seq1-R | GTGCCCACCCCAACCA |  |  |  |
| 29853_seq2-F | TTAGTCCAGAGGAAAGACCTAGCATT |  |  |  |
| 29853_seq2-R | TGACAAGGCTGAGGCTTAAATAAA |  |  |  |
| 29853_seq3-F | TGAGGGCAAATAGACTTCCTTGA |  |  |  |
| 29853_seq3-R | GCTCAGTTCAGCTGGTCTCTATCA |  |  |  |
| 30396_seq1-F | CTCACATCTGAAGAGAAAAAGACCTATATT | 4 | 180011013 | 180190536 |
| 30396_seq1-R | TGCTACCTGGGAATGTATTTTGTTAT |  |  |  |
| 30396_seq2-F | CAGCATTGTTGCATTTAGTCATCTT |  |  |  |
| 30396_seq2-R | CACTAGGGTCTCAACCTACTGATCAG |  |  |  |
| 30396_seq3-F | CTTACCAGGAGAAATCTATTAAGTCACAATA |  |  |  |
| 30396_seq3-R | TCCAACAAGTTACAAGGCATTGC |  |  |  |
| 32136_seq1-F | CCTCGCTCCATGCTATTCG | 9 | 114423074 | 114630399 |
| 32136_seq1-R | AAAAGCCGACACCTGCATTC |  |  |  |
| 32136_seq2-F | TTGAAAGCCTACCAAATGTTCTACA |  |  |  |
| 32136_seq2-R | TCATACGCCCTTGACAGTCACT |  |  |  |
| 32136_seq3-F | TGTATGTATAGGCAGTGCACAGAGACT |  |  |  |
| 32136_seq3-R | CCCCACCCCTATCCACTCA |  |  |  |
| 38232_seq1-F | TCATTGCCAGTGGAACATCATT | 7 | 153892821 | 154012562 |
| 38232_seq1-R | AAACAGATCAGTGTATCAACCCATTT |  |  |  |
| 38232_seq2-F | GCCCCATAATCAGACACCACAT |  |  |  |
| 38232_seq2-R | GAAACTCAGCCTGCCCAGAA |  |  |  |
| 38232_seq3-F | TGGCCTGAGACAGCAGAATAATC |  |  |  |
| 38232_seq3-R | GATGGCCGCGCACAATAC |  |  |  |
| 38689_seq1-F | CTGCTTGGGCTTCAAGAAAGA | 12 | 10696179 | 11095423 |
| 38689_seq1-R | TGAACGGCATGGCTTTCTG |  |  |  |
| 38689_seq2-F | TTCAAAAGCAGCTTTCGATCAG |  |  |  |
| 38689_seq2-R | TTTCAGAGCAAAGTCGTTCCTTAGT |  |  |  |
| 38689_seq3-F | TGCCTAGGTGGCTTATGGTAGAC |  |  |  |
| 38689_seq3-R | CACGCCCC TCTGCTCCTT |  |  |  |
| 43736_seq1-F | GCCATTTTTCCAGCATTCCTT | 16 | 75912405 | 76720656 |
| 43736_seq1-R | AAGGAAGTCACTGATAGGATAGCATACA |  |  |  |
| 43736_seq2-F | TTATCGGCGGAGTAGGTCAACT |  |  |  |
| 43736_seq2-R | CCTGGCTGCCTGGTACGTT |  |  |  |
| 43736_seq3-F | TTCAAGGTATAAAGACACGTAGAATAGCA |  |  |  |
| 43736_seq3-R | CCTGGAGGATGTTGGTCAAAA |  |  |  |
| 48028_seq1-F | GCCATGTGAGCAACACATTTATC | 20 | 60605746 | 60738767 |
| 48028_seq1-R | GCCCAGTTGTTCGTGTTGTCT |  |  |  |
| 48028_seq2-F | CATCCCTCCATTGTGCTGAA |  |  |  |
| 48028_seq2-R | CTCCCATGAGGTCCTGGAAA |  |  |  |
| 48028_seq3-F | CAGCTTAATGGCTTCGGTTAAAG |  |  |  |
| 48028_seq3-R | TGCCACCCGAGCTCAGA |  |  |  |
| 51553_seq1-F | GCTGCGGGACGTAAATGTTT | 1 | 2867639 | 3000446 |
| 51553_seq1-R | CCCCGTGTAAAAGCCATCTG |  |  |  |
| 51553_seq2-F | GGCTGCGGGCATGTTTAAT |  |  |  |
| 51553_seq2-R | ACCCCGAGCCATTGTCAA |  |  |  |
| 51553_seq3-F | TGGCCTTTTCCATGACTTGTG |  |  |  |
| 51553_seq3-R | GGAGCCTGCGTTTGGTCTAG |  |  |  |
| 55077_seq1-F | TGGTGATTCCTCATGCCCTATT | 13 | 88643956 | 88777785 |
| 55077_seq1-R | ACAGGCTCCCTGTAAAGGAAAAT |  |  |  |
| 55077_seq2-F | TCCAGAAGAATGTCCAGCTAAACTC |  |  |  |
| 55077_seq2-R | CATTTGGTCATGGCTTAGTGGAA |  |  |  |
| 55077_seq3-F | TTGAATCTGAGGTTTAGAATAATTCAGTTC |  |  |  |
| 55077_seq3-R | TGATGCTGCAGACGAAAATTG |  |  |  |
| 56884_seq1-F | CAGTAATAACAAGCAAATCAATCCTGTA | 5 | 120962504 | 121063944 |
| 56884_seq1-R | GTGTATGTTTTCCCCCCACTAAGT |  |  |  |
| 56884_seq2-F | GTTTCTAGGTAATTCACGGAACAGATT |  |  |  |
| 56884_seq2-R | CCTGACTTCTTGTTGAGGGAAAG |  |  |  |
| 56884_seq3-F | CCATTTGAGGCAAAGATTATTCATC |  |  |  |
| 56884_seq3-R | GCTGGACTATTTTGACCTGGTTTAA |  |  |  |
| 58203_seq1-F | TCATTTGACGCCTGGTTCCT | 6 | 4316609 | 4429349 |
| 58203_seq1-R | TGGAAATCCTGGGCCAATC |  |  |  |
| 58203_seq2-F | TCAGAGAAGGCTACACAACTTGACA |  |  |  |
| 58203_seq2-R | GTGGCACCCCAGGCTAAA |  |  |  |
| 58203_seq3-F | AACACCCCCAGCCACACA |  |  |  |
| 58203_seq3-R | TTAGCCCATCTCATGTTGCTCTT |  |  |  |
| 62859_seq1-F | CCTACCCCCAAGGGAAAGAG | 10 | 94144015 | 94253341 |
| 62859_seq1-R | TGATTCAGCTGCTGCCTTGT |  |  |  |
| 62859_seq2-F | GGCAGAAGGGCAGCAACA |  |  |  |
| 62859_seq2-R | TTCGTCCCCTCCAGGCTAGT |  |  |  |
| 62859_seq3-F | GCTGGCTCACCAAGGACTTAGA |  |  |  |
| 62859_seq3-R | GGCACACCAGCCTCTGCTA |  |  |  |
| 65882_seq1-F | TCTGCTTGCTTTAAGCATAGACATG | 8 | 100233363 | 100452817 |
| 65882_seq1-R | ATTGAGGTTTTAAACCCCAGAAAA |  |  |  |
| 65882_seq2-F | ACTGCATTCCAAACTGTTTGATATTT |  |  |  |
| 65882_seq2-R | CAGTGCACCTCACTGCTGATG |  |  |  |
| 65882_seq3-F | TGAAAGGGATGACTTAACACAACTG |  |  |  |
| 65882_seq3-R | CAAGTAGGGCATGACTGCTTCTT |  |  |  |
| 66304_seq1-F | CACCCAATGAATCCAAGATTGAA | 2 | 210646088 | 210816138 |
| 66304_seq1-R | TTGTCACCGCCAACTGATTTT |  |  |  |
| 66304_seq2-F | CCTGTAGCAGAAAATGCAAATCTTT |  |  |  |
| 66304_seq2-R | GTGGGATGATGCAAAAAATGG |  |  |  |
| 66304_seq3-F | AACAGATTTGGTCTTTGCTTTTCA |  |  |  |
| 66304_seq3-R | AGTTGCCTTTGATTAGAGGATGATTT |  |  |  |
| 68551_seq1-F | GAGACCCCGTGACATTTGAAG | 14 | 103663462 | 103775782 |
| 68551_seq1-R | GAGCTAAATGAGAATTGATCATTTTCC |  |  |  |
| 68551_seq2-F | CACATTTTGGACTGGGTTTCCT |  |  |  |
| 68551_seq2-R | TGACAGCAGCTGTTCCTGTCA |  |  |  |
| 68551_seq3-F | CCTTGGTAATTTGCGCAGACT |  |  |  |
| 68551_seq3-R | GACATGACAGACAGGAAGAGGAAA |  |  |  |
| 72960_Seq1-F | GCCCGGGATTTATAAGTGGAA | 8 | 30332064 | 30434997 |
| 72960_Seq1-R | CCTCCGAGTCCCGATTATCTG |  |  |  |
| 72960_Seq2-F | CTTTCCATTTCCCCATTGTGA |  |  |  |
| 72960_Seq2-R | AACAGCCCACCCTGGATGT |  |  |  |
| 72960_Seq3-F | TTTTGTTGTGACTTCAGCCTTGA |  |  |  |
| 72960_Seq3-R | CAGGGTGGCTTTCGGTTCT |  |  |  |
| 73394_seq1-F | TGGCAAAGCCGTCTAGCAT | 13 | 98462430 | 98563111 |
| 73394_seq1-R | GCGCTCATGTGATCCTTGGT |  |  |  |
| 73394_seq2-F | TGGCATGTTCTTTGCCTAGGA |  |  |  |
| 73394_seq2-R | GGGAGCCTGAAGAGCTCACA |  |  |  |
| 73394_seq3-F | CCCTTCCTCCAAAAACTTGCT |  |  |  |
| 73394_seq3-R | TTAGTATGCTCAGGTAGGACCAAGAA |  |  |  |
| 75350_seq1-F | CCCCGATTGGTTTCCCTTT | 12 | 122715134 | 122816846 |
| 75350_seq1-R | AGTAACCAAGGAAGGGTGTGTTG |  |  |  |
| 75350_seq2-F | CGAAAGCTTCGCCTATGTGTT |  |  |  |
| 75350_seq2-R | GCAAGCCCTAACTCTGCAAATAA |  |  |  |
| 75350_seq3-F | TGTGTTGGGCAGTGAGAGACA |  |  |  |
| 75350_seq3-R | ACCCCGTGCTGGCTTTC |  |  |  |
| 81793_seq1-F | TGCTCTCCCTGGCTTGAAGT | 6 | 68582233 | 68699466 |
| 81793_seq1-R | TGCAACCCACAAAACTAAATGC |  |  |  |
| 81793_seq2-F | CGGGCATTGACCGAACA |  |  |  |
| 81793_seq2-R | CAACACCGGTCACGGACTCT |  |  |  |
| 81793_seq3-F | CGACCCCCTAAGCCAGAATT |  |  |  |
| 81793_seq3-R | GATTCTTTCCTTTTCTCATTATCTTCTCA |  |  |  |
| 98250_seq1-F | AGGGTGAGTGTTCTGTAGCTGAGA | 4 | 106706770 | 106840090 |
| 98250_seq1-R | CCGCTTTGCCATCATCCT |  |  |  |
| 98250_seq2-F | CCAGGGCTCAGGCATTCA |  |  |  |
| 98250_seq2-R | GACGCTACAGCAACTTGGTTTG |  |  |  |
| 98250_seq3-F | CACCAAAATGCCATCAGGAA |  |  |  |
| 98250_seq3-R | ATGGGAAACAATTTCAGAACGTATT |  |  |  |
| 99615_seq1-F | GCAGAATTGTGCGGAAGCA | 15 | 66164151 | 66267941 |
| 99615_seq1-R | AGACCAATGCCCTACCAAGCT |  |  |  |
| 99615_seq2-F | CTTCATGTTTCCCCCACCTTT |  |  |  |
| 99615_seq2-R | TGGCACAGTGAGGAGCTCAA |  |  |  |
| 99615_seq3-F | GATTTCATGGCCAGCAGGTT |  |  |  |
| 99615_seq3-R | ATATTCCTCATGTGCCAACAACTC |  |  |  |
| 10388-seq1-F | TTAACTTTGTCACCGATGGCTTT | 18 | 9605186 | 9706942 |
| 10388-seq1-R | GGGATGGCATTTCCATTTCA |  |  |  |
| 10388-seq2-F | AGGAGCAGGCCCATTGG |  |  |  |
| 10388-seq2-R | GCCCTAGACTTTGCGATTGC |  |  |  |
| 10388-seq3-F | CACTCATTCAGCCAGACGGTATT |  |  |  |
| 10388-seq3-R | TCTTATTCATCCTGAAATCCCCTTA |  |  |  |
| 24605-seq1-F | CCAACCAAAGAATGTGCTAGACAT | 6 | 150025448 | 150189219 |
| 24605-seq1-R | TGGAGACAGACTGAAGCCATTAGA |  |  |  |
| 24605-seq2-F | GCCTAATGGAGTCACCCAAGTT |  |  |  |
| 24605-seq2-R | TCACTGGATGAGAGCCAGAAGA |  |  |  |
| 24605-seq3-F | GGAGCCCAAATTACGGAATCT |  |  |  |
| 24605-seq3-R | AGATGTTCCCTCAACATCATGTTG |  |  |  |
| 25850-seq1-F | AATGGCTACTGGTGCTCAAGGT | 5 | 46025150 | 46231639 |
| 25850-seq1-R | CAGTGATACAGTGAGCTAAGCCAAA |  |  |  |
| 25850-seq2-F | TTGTGGCTGCCTAGGACATG |  |  |  |
| 25850-seq2-R | TCTGAAATTCATCCTGGTCAACA |  |  |  |
| 25850-seq3-F | AGTGTCCAGTGTGGAGAGTTTAAGTTT |  |  |  |
| 25850-seq3-R | TCAGTGCCCATTGAACACACAT |  |  |  |
| 28298-seq1-F | GGAAGGGTGGTATGGATATGTTG | 7 | 24297920 | 24398499 |
| 28298-seq1-R | GAACTAGCTCTCGTGGAGAATCCT |  |  |  |
| 28298-seq2-F | CATCGCCCCTCCTGAACA |  |  |  |
| 28298-seq2-R | GGACAAGCCCACTAACTCCTTACTT |  |  |  |
| 28298-seq3-F | TGCCTATGTGGCTCCCAAA |  |  |  |
| 28298-seq3-R | GGAGAGGGCCACAGCTCAA |  |  |  |
| 32961-seq1-F | AAGGAATTTAATGCAGGTGAGTTGT | 14 | 44898497 | 44998947 |
| 32961-seq1-R | GCATTTCCTGCAGTTGTGGTT |  |  |  |
| 32961-seq2-F | GCCGGCTGAGAAGCTACTACTT |  |  |  |
| 32961-seq2-R | CCACCAAATCACTCCCATGAC |  |  |  |
| 32961-seq3-F | AAGACAATTCCAAAGGCAAGCT |  |  |  |
| 32961-seq3-R | AACGTCATTCGACTACCAAGTTCA |  |  |  |
| 42921-seq1-F | CCGTGTGACCTCTGCAGTACTC | 11 | 67268918 | 67493070 |
| 42921-seq1-R | ACACGGCGGAAAGATGACA |  |  |  |
| 42921-seq2-F | AAGCCTGATCCTGCCATGAA |  |  |  |
| 42921-seq2-R | TGCAAGCCAGCAGACAAATT |  |  |  |
| 42921-seq3-F | CGGAGCCTGTTCCAGATAGACT |  |  |  |
| 42921-seq3-R | CCGTCCAGCAAGGCTTCTC |  |  |  |
| 43802-seq1-F | TCAGGAATGGAGCCTGTTAGC | 5 | 124757596 | 128333414 |
| 43802-seq1-R | CCCACTCTCCTTCCCCTCTT |  |  |  |
| 43802-seq2-F | CCATGGTGAAAGATCAAGGAGAA |  |  |  |
| 43802-seq2-R | ATGGAGCAATGCTTGAGTGACTT |  |  |  |
| 43802-seq3-F | CATTTCAAAGACATGGCCTGTTC |  |  |  |
| 43802-seq3-R | GCACAGGGCAGCCAAGAC |  |  |  |
| 51358-seq1-F | ACACAGCATAGGGCAACATGAT | 2 | 185300143 | 185410935 |
| 51358-seq1-R | TGTCTCCCTACTGCTAGCCTAAGAA |  |  |  |
| 51358-seq2-F | GCTGCATTCACTTTGTGCATCT |  |  |  |
| 51358-seq2-R | TTGTTACTTTGATGATACTTTGGGATACTT |  |  |  |
| 51358-seq3-F | TGAACACAGAGCCAGGAACATAAC |  |  |  |
| 51358-seq3-R | TTTATTGCCTGAGGGAAACGA |  |  |  |
| 51477-seq1-F | AGGAGAAGCAACAGGCAATGA | 13 | 113421929 | 113561973 |
| 51477-seq1-R | TGACAGACCCCAGCAAGGA |  |  |  |
| 51477-seq2-F | AGAGCAGACAAGACTTTAAGCAGATTT |  |  |  |
| 51477-seq2-R | GGCAGCGCCTGCAGAT |  |  |  |
| 51477-seq3-F | CGGCCAAGGATGAGACCAT |  |  |  |
| 51477-seq3-R | TAACTCCTGGATCTTACTGCATTCG |  |  |  |
| 59352-seq1-F | CCGGAGTATTCCTCATCATCCT | 12 | 63798617 | 63932550 |
| 59352-seq1-R | TTGGCAATGCCATCATGTG |  |  |  |
| 59352-seq2-F | GGAGTGGGCACGCCATT |  |  |  |
| 59352-seq2-R | GCACGTTCCTATTGACACCAAA |  |  |  |
| 59352-seq3-F | TCAGTCAACGTGCCAGTACTCA |  |  |  |
| 59352-seq3-R | AGGCTGCCCTTCATGTCTTG |  |  |  |
| 66347-seq1-F | CACACACATA CACAAATCATACAGAACA | 2 | 177922589 | 178160574 |
| 66347-seq1-R | TTTTTGAGGAACTCCGACAGACT |  |  |  |
| 66347-seq2-F | TGGTTCCCTTGCACCTTTG |  |  |  |
| 66347-seq2-R | CACTGAGAAGGAACTGATTTGGAAA |  |  |  |
| 66347-seq3-F | CATCCAATTTCTTCCTGCTTTGA |  |  |  |
| 66347-seq3-R | CACATTAGGATCCAGGGAGACAA |  |  |  |
| 74114-seq1-F | TGGCCATGGACGTCATTG | 8 | 31077073 | 31290177 |
| 74114-seq1-R | CCCAACAGAAGGAATGTGCTTT |  |  |  |
| 74114-seq2-F | TCAGTTTTACATCATTCAGGTTCCA |  |  |  |
| 74114-seq2-R | AATGTCTTCCCGGATTGAAGAA |  |  |  |
| 74114-seq3-F | GGGCCATGGTCGGTTGA |  |  |  |
| 74114-seq3-R | TGTTGGTCCGCTTCCAACA |  |  |  |
| 75236-seq1-F | TTCATTATAACCCACAGCCAGAAA | 4 | 60730018 | 60838610 |
| 75236-seq1-R | TGGAGCCTAGAATTAAACAGAATGAC |  |  |  |
| 75236-seq2-F | GCTTTTTCAACATTTCAGTGTCATTG |  |  |  |
| 75236-seq2-R | GGAGAGTCACATAAGATGAGTATTAGAAACA |  |  |  |
| 75236-seq3-F | CAAAGGCCCAGTGGTTTCAG |  |  |  |
| 75236-seq3-R | TGTTTCAGGGACATCTGGGTAGA |  |  |  |
| 77264-seq1-F | CAATGTTCTGGGCTTTCCAATC | 8 | 90884475 | 90996342 |
| 77264-seq1-R | TTTGGAGGGCTGACCTGAGA |  |  |  |
| 77264-seq2-F | GCCAAGGGAGTTCTGATGCA |  |  |  |
| 77264-seq2-R | TCCAGCAATGGCCCAAAT |  |  |  |
| 77264-seq3-F | TTCGTGTTCTGGAAGATGAAGAGT |  |  |  |
| 77264-seq3-R | AATTTATACTACCACATGGAAAGAAGGTT |  |  |  |
| 77777-seq1-F | GGCAGCTCTGTCCCTTTGG | 1 | 204767063 | 204911423 |
| 77777-seq1-R | TGCAATCTGTGCATTCCATGT |  |  |  |
| 77777-seq2-F | TCCGGAGTGAAGCCCATACT |  |  |  |
| 77777-seq2-R | GGCCAGGTGCTGCAGAGT |  |  |  |
| 77777-seq3-F | TGGCTTCTTTCCCAGTTCTTAAA |  |  |  |
| 77777-seq3-R | AGACTGGAGTTCTCAAGCCATTG |  |  |  |
| 88463-seq1-F | CACAGGATTAAAACGGGTCATTAA | 16 | 34054852 | 34162068 |
| 88463-seq1-R | TCGGAGTTGTGTTTTATTTTGAACTT |  |  |  |
| 88463-seq2-F | CAGCGCCTCTGTTCTTTAAACC |  |  |  |
| 88463-seq2-R | TTCCGTCGGCAAAAGCA |  |  |  |
| 88463-seq3-F | CTGGGTTCAAAACCTTATCAGAAGA |  |  |  |
| 88463-seq3-R | AGCGCTGAGACCATACAATGC |  |  |  |
| 89844-seq1-F | GTAAGGGTGAGAATGGCTCTCTTT | 2 | 212852308 | 212955735 |
| 89844-seq1-R | GGGTTTGCACTTCATGGATGT |  |  |  |
| 89844-seq2-F | CAGCCTGAAGGGTGGTAATGA |  |  |  |
| 89844-seq2-R | TGAGCCTGGGAGGGAAAAA |  |  |  |
| 89844-seq3-F | AAACAGTTCAACTCTTCAACCCCTAT |  |  |  |
| 89844-seq3-R | TATCCAGGAAAAATCTTCGCTTTC |  |  |  |
| 92868-seq1-F | GAGGTCCCGGCATTTACTCA | 4 | 3565190 | 3855607 |
| 92868-seq1-R | TCCATGGGTCCCTGTCTGA |  |  |  |
| 92868-seq2-F | CGTGTGCGAGGCTGTTTACA |  |  |  |
| 92868-seq2-R | TCGAGGAGCTCTCAGCAGTCT |  |  |  |
| 92868-seq3-F | CAGCGGTAAAGGTGGCAAAG |  |  |  |
| 92868-seq3-R | CTGCCTGTGGGCGTCTATTT |  |  |  |
| **Three common CNV regions** | | | | |
| CNP2157-F | CCACTATGTGCATTCTGACACCTT | 16 | 22465433 | 22612022 |
| CNP2157-R | TCCCAGTTAAAGTTGTGTCTGAAGAAAA |  |  |  |
| Reporter sequence | ACGGGACTCACCAAAAGAAAA |  |  |  |
| CNP1293-F | CCATCAAAAACACTTTTATTCAACTATGCA | 8 | 39354760 | 39506122 |
| CNP1293-R | AACACAATCAGGTTGCATTTTAAAGCT |  |  |  |
| Reporter sequence | TTTCCCTTTCCTCCACTGATTCTA |  |  |  |
| CNP2057-F (Hs03912427_cn)* |  | 15 | 19803370 | 20089386 |
| CNP2057-R  (Hs03912427_cn) |  |  |  |  |
| Reporter sequence | TGTGCACAGAGACACAAAATTCCTG |  |  |  |

***This probe is pre-designed by ABI, so the sequence is unavailable.**

**Supplementary Figure S1, CNV Recovery Rates**

The recovery rate was depicted against the frequency of CNVs in HapMap samples, which were spanned by more than 20 markers. The x axis is the frequency of CNVs. The y axis represents the recovery rate of Birdsuite. The average recovery rate of CNVs decreased when the frequencies of the CNVs increased.


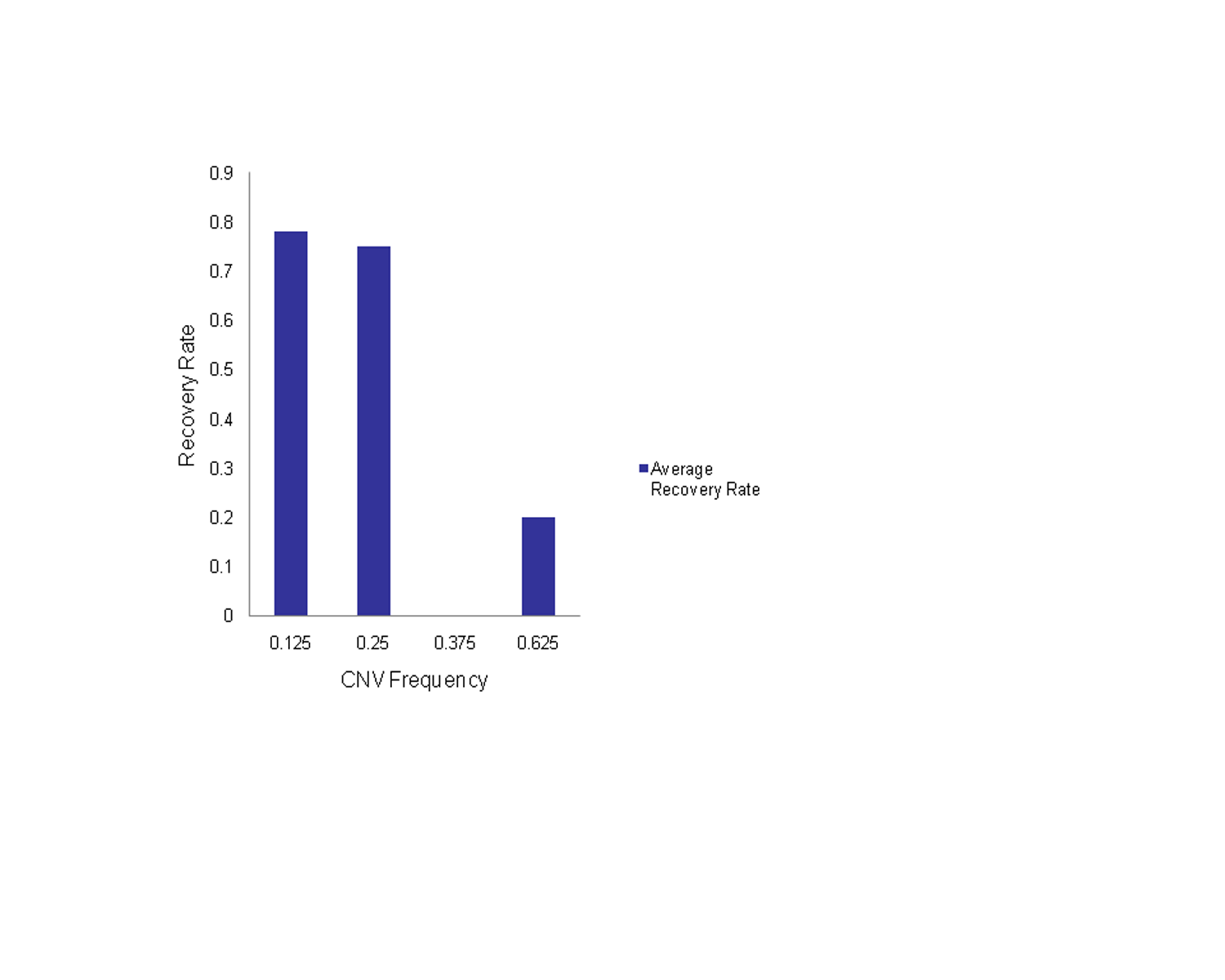

Supplement: File S1 — Includes Tables S1 to S3 and Figure S1. (0.42 MB DOC) [file pone.0014511.s001.doc]
